# Mitral leaflet aneurysm and perforation in fulminant infective endocarditis: Insights from real-time three-dimensional echocardiography

**DOI:** 10.21542/gcsp.2026.18

**Published:** 2026-04-30

**Authors:** Miguel Vicente, Catarina David, Fidel Manuel Caceres-Loriga, Humberto Morais

**Affiliations:** 1 Cardiology Department, Coimbra Hospital and University Centre, Portugal; 2 Cardiovascular and Thoracic Centre –Clínica Girassol, Luanda, Angola; 3 Agostinho Neto University Faculty of Medicine, Luanda, Angola; 4 Doctors HealthCare, Florida, USA; 5 Hospital Militar Principal/Instituto Superior, Luanda, Angola

## Abstract

Infective endocarditis is a life-threatening condition with high morbidity and mortality, particularly in low- and middle-income countries. Amongst causes, enterococcal IE accounts for a substantial proportion of cases, with *E. faecalis* as the predominant species (90%). We report a 59-year-old man with arterial hypertension and type 2 diabetes mellitus who presented with asthenia, low-grade fever, and osteoarticular pain. Laboratory evaluation revealed severe anemia, leukocytosis, and thrombocytopenia. A new holosystolic mitral murmur prompted echocardiography, which identified vegetation, aneurysm, and perforation of the anterior mitral leaflet with severe regurgitation. Blood cultures confirmed *E. faecalis*, and appropriate antibiotic therapy was initiated. Despite rapid intervention and intensive care, the patient developed acute heart failure and died within 23 days of admission. This case illustrates the fulminant potential of *E. faecalis* IE complicated by complex mitral valve destruction, and underscores the importance of early recognition, real-time 3D echocardiography, and prompt multidisciplinary intervention in high-risk presentations.

## Introduction

Infective endocarditis (IE) is a potentially fatal condition and remains a serious public health problem, particularly in low- and middle-income countries^1, 2^. Its global incidence is estimated at approximately 13.8 cases per 100,000 individuals per year, accounting for around 67,000 deaths annually worldwide^1, 3^.

Enterococcal IE represents the third most common cause of this disease, ranking behind infections caused by streptococci and staphylococci^4^. *E. faecalis* is the predominant etiologic agent, responsible for approximately 90% of cases^1^ and despite advances in prevention, diagnosis, and treatment, morbidity and mortality remain high^1, 2^.

The infectious process usually begins with damage to the valvular endothelium, often associated with predisposing factors such as prosthetic valves or cardiac implantable devices^1–3^. These conditions promote areas of endothelial injury, allowing microorganisms—predominantly bacteria (≈80%), but also fungi or other pathogens—from cutaneous, oral, gastrointestinal, or genitourinary sources to adhere. Once attached, these pathogens colonize and proliferate, leading to inflammation, tissue destruction, and valvular dysfunction through mechanisms such as vegetations, abscess formation, and ultimately leaflet perforation and destruction^2, 5^.

Diagnosis is based on the modified Duke criteria (updated 2023), combining clinical, microbiological, and imaging findings. The clinical presentation is highly variable, often leading to diagnostic delay. Fever ≥38 °C, although present in approximately 77% of cases, may be absent or without an apparent source^1, 4^. Positive blood cultures are crucial for identifying the causative pathogen, ideally confirmed in at least two blood samples drawn from different sites, or, in the case of Coxiella burnetii, from a single positive culture or phase I IgG antibody titre >1:800^1, 2^. When blood cultures are negative, culture-negative IE should be considered, with prior antibiotic use representing the most common explanation. Transthoracic and transesophageal echocardiography remain the primary imaging modalities for vegetation detection and diagnostic confirmation^1, 2^.

Management of IE requires a multidisciplinary approach to define individualized strategies based on the causative agent, disease severity, and complications^1, 2, 6^. These complications may be cardiac—such as aneurysms, pseudoaneurysms, abscesses, leaflet perforation, or prosthetic valve dysfunction—or extracardiac, including neurological, splenic, renal, and musculoskeletal manifestations, with each carrying distinct prognostic implications^1^.

Early detection of such complications maybe be supported by advanced imaging methods, including cardiac computed tomography (CT), whole-body angiography, brain magnetic resonance imaging (MRI), labeled leukocyte scintigraphy, and 18F-fluorodeoxyglucose positron emission tomography (18F-FDG PET/CT)^1, 2, 7, 8^. Continuous clinical and imaging surveillance is essential to identify the threshold for surgical intervention, including emergency valve surgery when haemodynamic compromise or uncontrolled infection is present^1, 7^.

We report a case of enterococcal IE complicated by concurrent mitral valve aneurysm, vegetation, and perforation —a rare structural combination —that proved fatal despite prompt multidisciplinary intervention.

### Case Report

A 59-year-old man with arterial hypertension and type 2 diabetes mellitus, irregularly followed in cardiology consultations, presented to the emergency department with a five-day history of asthenia, low-grade fever, and osteoarticular pain, with marked deterioration in the 24 h prior to admission. He appeared chronically unwell, with signs of poor nutritional status, pale mucous membranes, fever (38.1 °C), and tachycardia. The remainder of the physical examination was unremarkable. Antipyretic therapy was administered with temporary improvement.

Laboratory tests showed severe anaemia (hemoglobin 5.4 g/dL), leukocytosis (14,030/µL) with neutrophilia, and thrombocytopenia (34,400/µL), with no identifiable source of infection. Chest X-ray demonstrated an increased cardiothoracic ratio, consistent with cardiomegaly. Electrocardiography showed sinus rhythm, first-degree atrioventricular block, and voltage criteria for left ventricular hypertrophy.

Investigations for an alternative source of the severe anaemia included upper gastrointestinal endoscopy, which was unremarkable; colonoscopy was attempted but not completed due to poor patient tolerance. On day 15 of admission, two further febrile episodes were recorded (38.3 °C and 38.1 °C). Crucially, clinical re-examination at this point revealed a new grade V/VI holosystolic mitral murmur radiating to the axilla —a finding absent on initial assessment. Cardiology review was urgently requested. In the context of persistent fever, new-onset mitral regurgitation, and severe anaemia without an identified source, IE was suspected. Three sets of blood cultures were drawn from separate sites, and empirical therapy with ampicillin and ceftriaxone was commenced in accordance with current guidelines.

**Figure 1.**
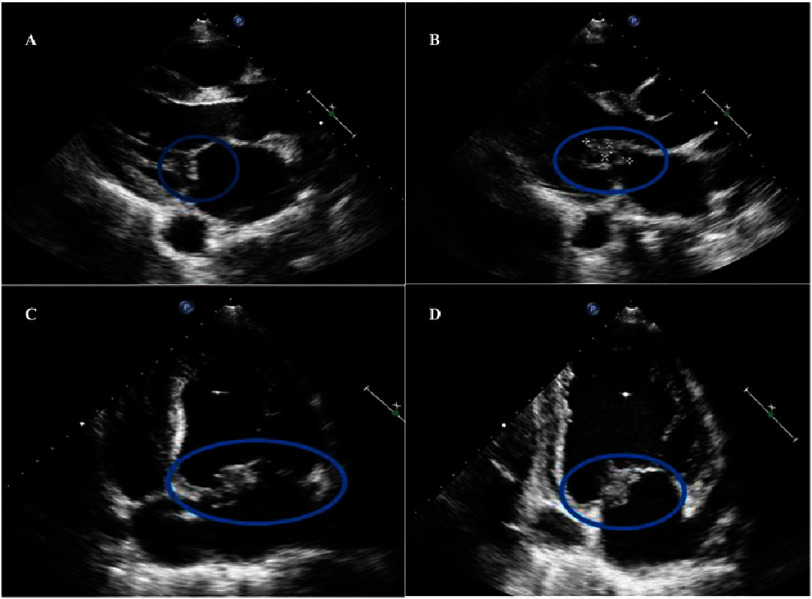
(A and B) Transthoracic echocardiogram, parasternal long axis view showing thickening of the leaflets and a mass attached to the anterior leaflet of the mitral valve measuring 2.94 × 0.88 cm. (C and D) Apical 4 and 2 chambers; mass on the anterior leaflet.

**Figure 2.**
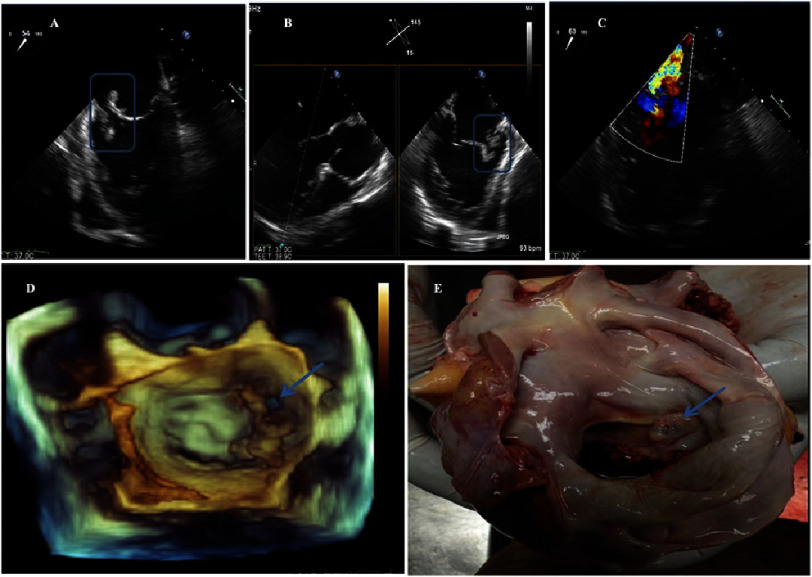
(A and B) Transoesophageal echocardiogram: biplanar images of the mid-oesophagus at 54° and 145° (right-left), showing an aneurysm and vegetation attached to the anterior leaflet of the mitral valve. (C) Severe mitral regurgitation due to perforation (Carpentier I). (D) Three-dimensional echocardiogram, perforation of leaflet A3 (blue arrow). (E) Cardiac autopsy specimen, left atrial view, showing perforation of the anterior mitral leaflet (blue arrow).

Bedside transthoracic echocardiography revealed an echogenic mass consistent with vegetation attached to the anterior mitral leaflet (A2/A3 segment) measuring 2.94  × 0.88 cm (Figure 1). Subsequent 2D and real-time 3D transesophageal echocardiography confirmed aneurysm and perforation of the anterior mitral leaflet (A3) with severe mitral regurgitation (Figure 2).

Because of acute heart failure (NYHA functional class IV), the patient was transferred to the cardiac intensive care unit for stabilization and immediate preoperative preparation. Blood cultures were positive for *Enterococcus faecalis*, and antibiotic therapy was adjusted (ampicillin 12 g/day and ceftriaxone 4 g/day). Despite intensive management, the patient died on day 23 of admission. Post-mortem examination confirmed the echocardiographic findings, including mitral leaflet aneurysm, perforation, and extensive vegetation (Figure 2E).

**Figure 3.**
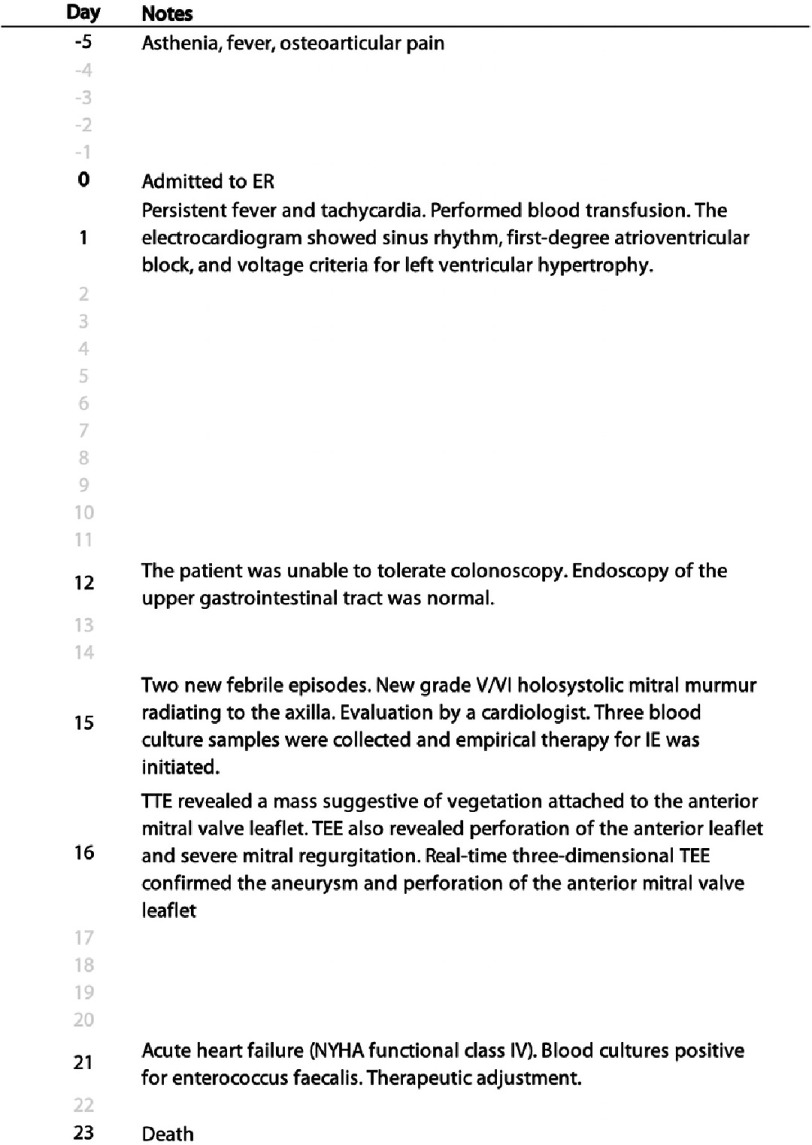
Timeline of the case.

A timeline of the case is presented in Figure 3.

## Discussion

IE is a severe and potentially fatal infection that predominantly affects cardiac valves. Given the high in-hospital mortality—up to 30%—early diagnosis and treatment are critical^1–3, 6^. The epidemiological profile has changed in recent decades, with an increasing number of cases related to intracardiac devices, prosthetic valves, and intravenous drug use, while rheumatic fever remains relevant in low-income regions. Prompt diagnosis and early therapy are essential to prevent complications such as heart failure and systemic embolization.

*E faecalis* accounts for approximately 90% of enterococcal IE cases and presents significant treatment challenges due to its antimicrobial resistance profile and the low bactericidal activity of *β*-lactams alone^9^, requiring around six weeks of synergistic antibiotic therapy^1, 2, 8^.

Guidelines recommend synergistic regimens, traditionally ampicillin with gentamicin. In elderly patients or those at risk of nephrotoxicity, the ampicillin–ceftriaxone combination has shown comparable clinical efficacy with a more favourable nephrotoxicity profile, as demonstrated by Fernández-Hidalgo et al.^9^. The choice of regimen should be guided by susceptibility testing, serial blood cultures, and close clinical monitoring.

In this case, after blood cultures were obtained, empirical therapy with ampicillin and ceftriaxone was initiated based on clinical suspicion of enterococcal IE. The 2023 ESC guidelines recommend broader empirical coverage pending culture results; however, the ampicillin–ceftriaxone regimen was selected in view of the patient’s nephrotoxicity risk profile, local resistance patterns, and resource availability.

Initial laboratory findings were consistent with severe sepsis, with the degree of anaemia and thrombocytopenia suggesting additional consumptive or haemolytic processes. Diagnosis was established on the basis of two major modified Duke criteria: echocardiographic evidence of vegetation and positive blood cultures yielding *E. faecalis*^10^.

Transthoracic echocardiography is the recommended first-line modality; however, transesophageal echocardiography is essential when structural complications are suspected, offering superior resolution of valvular anatomy and perivalvular extension^1^. Advanced imaging modalities such as cardiac CT and 18F-FDG PET/CT are recommended for detecting perivalvular complications and are incorporated into the 2023 modified Duke criteria^3, 7, 10, 11^. In this case, real-time 3D echocardiography enabled detailed visualization of the vegetation, aneurysm, and anterior mitral leaflet perforation, providing critical information for surgical planning. The coexistence of vegetation, aneurysm, and leaflet perforation represents a particularly destructive phenotype of mitral IE, associated with accelerated haemodynamic compromise and high surgical urgency^7^.

The patient’s rapid deterioration to NYHA class IV heart failure and the emergence of a new holosystolic murmur suggested an acute structural complication. Both ESC and AHA guidelines recommend emergency surgery in such cases to prevent circulatory collapse and reduce mortality^1, 12^. Despite clear guideline-based indications for urgent surgery, intervention was not performed due to clinical and contextual factors, including high perioperative risk and lack of immediate access to cardiac surgery. This reflects a common challenge in resource-limited settings, where guideline adherence is often constrained by infrastructural barriers.

Delayed access to diagnosis and treatment in such settings is associated with higher mortality, emphasizing the need for improved care pathways. While guideline-directed preventive strategies exist, their impact is limited when access to definitive care is restricted. *E. faecalis* endocarditis has been increasingly associated with underlying colonic pathology, including colorectal neoplasia^13^.

In this case, incomplete lower gastrointestinal evaluation is a notable limitation, as occult colorectal pathology, including malignancy, is a recognised portal of entry in *E. faecalis* bacteraemia and may have gone undetected. Current guidelines recommend complete colorectal evaluation in all cases of *E. faecalis* IE, given the established association with colorectal neoplasia.

### What have we learned?

• IE is a serious infection with a high mortality rate.
• Immediate diagnosis and early accurate treatment have an impact on reducing serious complications.
• Systemic and cardiac complications must be addressed early to reduce mortality.
• Cardiovascular imaging is a powerful ally in the diagnosis and monitoring of IE.
• Limited access to advanced imaging, cardiac surgical capacity, and specialist multidisciplinary care in low- and middle-income countries is a significant barrier to optimal management of IE.

## Author statement

Study design: HM and FMCL / Data collection: HM, CD, MV Writing of the manuscript: HM, CD, MV / Revisions and approval of the final manuscript: All authors

## Conflicts of interest

The authors declare no conflicts of interest and no specific funding sources for this work.

## Consent

Written voluntary informed consent was obtained from the patient’s family. The study is in accordance with ethical principles.
